# Stimulating Future-Oriented Thinking and Goal-Achievement Through the Future Self Using Virtual Reality and a Smartphone App: Randomized Controlled Trial

**DOI:** 10.2196/84420

**Published:** 2026-05-13

**Authors:** Jean-Louis van Gelder, Tiffany Tettero, Aniek Siezenga, Esther Mertens

**Affiliations:** 1 Department of Criminology, Max Planck Institute for the Study of Crime, Security and Law, Günterstalstraße 73, Freiburg, 79100, Germany, 49 761 7081 324; 2 Institute of Education and Child Studies, Leiden University, Leiden, The Netherlands; 3 Netherlands Institute for the Study of Crime and Law Enforcement, Amsterdam, The Netherlands

**Keywords:** future self-identification, future orientation, future self-continuity, intervention, mental time travel, goal achievement, virtual reality, smartphone app

## Abstract

**Background:**

Research suggests that identification with one’s future self—encompassing vividness, connectedness, and valence—plays a key role in motivating future-oriented choices and goal pursuit. Interventions aiming to strengthen future self-identification have been shown to reduce maladaptive behaviors and promote well-being, but traditional approaches often rely heavily on imagination. Emerging technologies offer novel opportunities to make the future self more vivid and tangible, potentially reducing cognitive burden, and enhancing intervention effectiveness.

**Objective:**

This randomized controlled trial evaluated the effectiveness of a digital intervention on the following primary outcomes: future self-identification, future orientation, consideration of future consequences, self-defeating behavior, goal commitment, and goal achievement.

**Methods:**

In this parallel, 3-arm randomized controlled trial, 321 first-year students from a large public university in the Netherlands were randomized (1:1:1) with blocks of 9 to a smartphone app–based future-self intervention, an immersive virtual reality (VR) version of the same intervention, or an active goal-setting control condition. Participants assigned to the smartphone or VR condition engaged with the intervention over a 3-week period, during which they interacted with their 10-year-old self, their future self. Participants in the control condition received no further support. Each of the 3 conditions consisted of 107 participants, who were all included in the analyses following the intention-to-treat principle. Due to the nature of the intervention, blinding was not possible.

**Results:**

Compared to the goal-setting control condition, both intervention conditions yielded significant short-term improvements on all 3 aspects of future self-identification (smartphone: *d*_vividness_=0.49; *d*_valence_=0.44; *d*_connectedness_=0.43; VR: *d*_vividness_=0.35; *d*_valence_=0.44; *d*_connectedness_=0.43), and a small decline in vividness (smartphone: *d*=−0.36; VR: *d*=−0.23) and connectedness (smartphone: *d*=−0.36; VR: *d*=−0.32) at 6-month follow-up. Additionally, the intervention buffered declines in future orientation during the study period (smartphone: *d*=.16; VR: *d*=0.18), and VR delivery led to significantly higher weekly goal achievement (*d*=0.88). However, effects on future orientation were not sustained over time (smartphone: *d*=−0.18; VR: *d*=−0.25), resulting in similar levels across conditions at 6-month follow-up. Furthermore, no significant effects emerged for other primary or secondary outcomes, such as self-defeating behavior (smartphone: *d*_overall_=−0.10; VR: *d*_overall_=−0.03), impulsivity (smartphone: *d*_overall_=−0.07; VR: *d*_overall_=−0.09), or academic performance (η^2^_partial_=0.00). No adverse events were reported in any of the conditions.

**Conclusions:**

This randomized controlled trial innovatively compares smartphone and immersive VR future-self interventions. Findings suggest that digital interventions leveraging visual and interactive representations of the future self can strengthen future self-identification and future orientation, and support (short-term) goal pursuit. Together, they highlight the potential of scalable digital future self approaches for educational and preventive health contexts.

## Introduction

### Background

Considering the future is a key component of psychosocial functioning and a ubiquitous part of mental life [1,2]. Individuals who are more future-oriented tend to make more informed trade-offs between the short-term and long-term consequences of their decisions [3] and are more likely to set goals they aspire to achieve over time [4]. This orientation is also linked to various beneficial outcomes, including heightened feelings of competence, better health, increased savings, and stronger academic performance [4,5]. In contrast, individuals who are more present-focused more often act on impulse and display a preference for immediate gratification [6,7]. Such a focus has been associated with behaviors that offer short-term benefits, but often entail severe long-term costs—that is, are self-defeating—such as delinquency, gambling, and substance use [6,8]. Thus, enhancing future-oriented thinking could contribute to psychosocial functioning in important ways. Here, we examine the effectiveness of an intervention aiming to increase future-oriented thinking and goal achievement by strengthening identification with the future self, using a smartphone app and immersive virtual reality (VR) as delivery technologies.

### A Future-Oriented Mindset and the Future Self

Research leveraging the future self to stimulate future-oriented behavior and successful goal pursuit is premised on the idea that the degree to which individuals consider temporally distant outcomes is related to how strongly they identify with, and feel connected to, their future self [9]. Tendencies to disregard or downplay possible long-term consequences of current actions and decisions are believed to result from an inability to project one’s self into the future [10]. Rather than emphasizing how current behaviors may carry (negative) future consequences in a more generalized way, which is unlikely to resonate with those who have a strong here-and-now orientation, this approach emphasizes the idea of a shared fate between the present and the future self [11]. Just as people experience varying degrees of closeness to others—and these differences affect how willing we are to make sacrifices on their behalf—varying degrees of identification with our future selves can shape to what extent we act in ways that benefit those future selves.

Experiencing a stronger sense of identification with one’s future self is assumed to contribute to a greater awareness that the possible costs of current actions will ultimately be borne by oneself, even if they materialize only at a later point in time, and also makes the burden of such costs more salient [10]. Conversely, it also promotes the realization that the potential benefits of current effort and sacrifice will get to be enjoyed by oneself, even if such benefits are reaped only in the future. Thus, stronger identification with one’s future self should make it more likely that the interests of this self are factored into decisions made in the present [12].

In support of these assumptions, empirical studies have shown that enhancing identification with the future self promotes positive behaviors, such as increased physical activity [9], addiction recovery [13], and savings behavior [12,14], while reducing negative behaviors, such as delinquency [15,16], cheating [17], temporal discounting [18], and procrastination [19]. For example, in a study using a large nationally representative sample in the United States, Hershfield et al [14] found that individuals with a higher perceived connection to their future self demonstrated better financial well-being and more robust saving behavior. A study among convicted offenders showed that exposure to their future self using a VR paradigm resulted in a decrease in self-defeating behavior [16].

Furthermore, identification with the future self has recently been found to be a protective factor in mental health. Research involving adolescents has shown that a lack of possible selves or negative valence toward the future self is a key characteristic of depressive symptoms [17]. Other work found that people struggling with depression or suicidal ideation often describe their future selves as distant, dissimilar, and emotionally disconnected—a pattern reflecting low future self-identification [18,19]. Strengthening identification with the future self could therefore have cascading effects in different domains. Recent work has started leveraging the future self as an intervention mechanism for improving people’s ability to think about the future and to reduce symptoms of depression and suicidal ideation [20]. In short, interventions that target the future self may offer transdiagnostic benefits.

### Future Self-Identification as Vividness, Connectedness, and Valence

Future self-identification (also referred to as future self-continuity in the research literature) is often conceived as a tripartite structure consisting of the extent to which individuals can clearly and concretely imagine and describe their self in the future (vividness), how similar and connected they feel to it (connectedness), and the positive or negative affect they associate with it (valence) [21,22]. Future self vividness and connectedness, in particular, have been shown to predict intertemporal choice; however, they are believed to operate through different mechanisms.

Vividness is related to psychological distance, which shapes how events are mentally represented. Events anticipated in the distant future tend to be construed in more abstract and decontextualized terms and with less detail, making them less likely to prompt action [23,24]. Events perceived as temporally near, in contrast, are represented more concretely and are more likely to motivate behavior. One important mechanism accounting for this is that more concrete representations enhance the perceived likelihood and immediacy of those events; the ability to vividly imagine specific, personal future scenarios can make distant goals feel more immediate and is thus more likely to prompt action [2,25]. Furthermore, vivid mental representations also intensify the emotional salience of potential outcomes, which can influence behavior over and above the subjective likelihoods of those outcomes [6,26]. Thus, enhancing the vividness of the future self reduces the psychological distance to the current self and increases the emotional salience of remote outcomes and thereby motivates goal-directed action.

Future self connectedness denotes the degree of overlap between the current and future selves one experiences in terms of factors such as shared goals, values, identity, and personality characteristics [22,27]. People experiencing high levels of connectedness are more likely to view their future self as an extension of their current identity. In contrast, people who experience only a weak sense of connection with their future self may regard it as distinct and unfamiliar, akin to perceiving a stranger [12,28]. The sense of connection people experience with their future self is consequential, as it affects whether people will act in its best interest (vs acting only with the well-being of their present self in mind). That is, similar to how individuals experience varying degrees of psychological connectedness to others—which in turn influences their willingness to make sacrifices on others’ behalf—variability in one’s sense of connection to the future self can predict the extent to which they engage in behavior that serves the interests of their future selves [10].

Valence of the future self regards the positive and negative affect people associate with it. Although it stands to reason that people with more positive views of their future self are likely to invest more in their future, and positive evaluations of one’s future self have also been empirically linked to current self-esteem, mental health, and overall well-being, the empirical relationship between future-self valence and future-oriented behaviors is not well established [10]. Research on episodic future thinking also shows mixed findings regarding the relation between valence and temporal discounting, an indicator of short-term thinking [29].

Prior research has tended to either use an index measure of future self-identification that includes all three aspects—vividness, connectedness, and valence—or has focused on only a single aspect, leaving out the other two. Index measures are unable to identify which of the three aspects is driving the effect on outcomes and may mask meaningful relations between specific aspects and outcomes. Research focusing on only one aspect leaves the possible effects of the other two unaccounted for. In this study, we include all three aspects of future self-identification and examine the effects of the intervention on each of them separately.

### Modes of Delivery: Smartphone App Versus VR

Traditional interventions to promote future-oriented thinking and behavior often rely heavily on people’s imaginative abilities and use exercises that can carry a significant cognitive burden, such as asking people to imagine a possible future event [30] or to write a letter to their future self [31]. Visual and immersive technologies, such as smartphone apps and VR, provide not only visual support through graphic renderings of a future self but also allow for a sense of interaction with, and, in the case of VR, the possibility of embodiment of, the future self. Hence, such interventions can reduce cognitive load and the dependency on people’s imaginative abilities.

Aside from their shared ability to provide interactive visual content, as delivery methods, apps and VR also have several distinct characteristics, and they differ in the way users can interact with them [32]. Smartphones tend to be integrated in people’s daily lives, which provides the opportunity to deliver an intervention in their natural environment through frequent exposure and interaction [33,34]. Users can access the intervention content wherever and whenever they want, as they carry the intervention with them “in their back pocket.”

VR, in contrast, offers the opportunity to provide participants with highly immersive experiences tailored to the goals of an intervention [32]. For example, VR allows users to embody avatars that differ meaningfully from their own physical or identity characteristics in perceptually convincing ways [35]. Such alterations in avatar design can unconsciously influence users’ perceptions or behaviors, even when they remain consciously aware of the artificial nature of the virtual setting, and thereby change attitudes and behaviors [36,37]. To capitalize on this affordance, in this study, participants embody avatar versions of their future (and current) selves in a virtual environment.

### This Study

The goal of this study was to evaluate the effectiveness of an intervention developed in the context of the FutureU research program. The intervention aims to foster future-oriented thinking and future self-identification through three broad strategies: (1) prompting participants to imagine their future and desired goals, (2) encouraging reflection on their future self, and (3) enabling interaction with digital renderings of this temporally remote self. These renderings are intended to instill a more vivid image of the future self, to encourage participants to think about their future self in daily life, and to stimulate a sense of connection to it. Through various assignments, participants are exposed to, and interact with, a digital version of their future self 10 years from now (see Table 1 for a description of the different intervention modules).

**Table 1.** Description and features of the 3 intervention modules (future self-identification, future self-perspective, and goal setting and achievement), including underlying theory and core features implemented across conditions^a^.

Module	Theory	Core features smartphone app	Core features VR^b^ sessions
Future self-identification			
Stimulating vividness, familiarity, and identification with the future self	Exposure to and vividness of the future self increases future orientation [38]. Additionally in smartphone intervention Incremental personality theory: The belief that personality can change over time can reduce problematic behaviors [39]. People’s willingness to change on personality traits in socially desirable ways increases after feedback on their current trait levels [40].	Complete personal profile of the future self (eg, work experience, skills, and accomplishments). Current scores on personality traits with an indication of norm scores. Short animation with psychoeducation that personality can change over time. Set scores of personality traits of the future self.	Time travel portal facilitating mental time traveling in order to “prelive” events. Embodiment of the avatar representing future self, bolstering vividness of and identification with the future self. Interview future self about personal profile (eg, work experience, skills, and accomplishments). A grid containing participants’ answers showing a personal profile of the future self.
Future self-perspective			
Cultivate future-oriented choices and increase self-insight by distanced perspective taking, aiming to stimulate attitudes and behaviors favoring the future self	People make more future-oriented choices (1) for others (ie, Solomon’s paradox) [41], (2) when they have a vivid perception of the future self [38], and (3) when they can psychologically or temporally distance themselves from the situation (ie, construal level theory) [23]. Wise reasoning is enhanced with third-person self-reflection [42].	Short animation clip with psychoeducation that people make more future-oriented choices when they distance themselves from the situation, and when they think about the long-term consequences. Time portal to take a future self-perspective for giving advice. Participants address themselves in the third person. Future self-interaction portal: The future self emphasizes that personality can change over time and stimulates decision-making with the future self in mind.	Verbal psychoeducation that people make more future-oriented choices when they distance themselves from the situation and when they think about the long-term consequences. Ask the future self for advice on a challenge in the study domain and on a freely chosen domain. Switching perspectives is used to mimic a conversation and facilitates clarification of the challenge or problem and giving advice. A grid containing participant’s answers showing the posed challenges and the future self’s advice.
Goal setting and achievement			
Bolster goal setting and achievement by teaching a growth mindset and mental contrasting and implementation intentions	Growth mindset: The belief that people’s abilities can develop over time. This mindset aids engagement in thoughts and behaviors to work toward goals [43]. Mental contrasting and implementation intentions [44]: A method in which the desired future is contrasted with the current reality and then reflected upon obstacles in the way of attaining the desired future. Subsequently, a plan is formulated to implement behaviors to overcome obstacles, that is, implementation intentions, in the format: If situation X, then I will do Y.	Short animation clip with psychoeducation that abilities can develop over time. Short animation clip explaining mental contrasting and implementation intentions. Practice with mental contrasting and implementation intentions to work toward goals via filling in a scheme. Writing a letter to the future self with goals. Future self-interaction portal: The future self stimulates taking the perspective of the future self (in decision-making and in goal achievement) and provides a guided episodic future thinking exercise.	Verbal psychoeducation that abilities can develop over time. Practice mental contrasting and implementation intentions via interviewing the future self. A grid containing participants’ answers providing a scheme of goals, obstacles, and plans to overcome the obstacles.

a Reprinted from the protocol paper of the research project [45].

b VR: virtual reality.

We examined intervention effects on future orientation, goal achievement, and self-defeating behaviors, comparing the smartphone and VR experimental conditions to an active, goal-setting control condition. Specifically, we assessed proximal effects on the 3 aspects of future self-identification (ie, vividness, connectedness, and valence), as well as distal intervention effects on primary outcomes (ie, future orientation, consideration of future consequences, self-defeating behavior, goal commitment, and goal achievement) and secondary outcomes (ie, self-efficacy, academic performance, and impulsivity). In case of significant intervention effects, we compared the 2 delivery methods (ie, app vs VR) with each other to establish whether one generated stronger effects than the other. We hypothesized that the intervention would enhance future self-identification and result in higher scores on the primary outcomes. No specific hypotheses regarding the most effective delivery method were formulated.

## Methods

### Trial Design and Procedure

The intervention was examined by means of a parallel randomized controlled trial, with three conditions: (1) a smartphone condition in which participants set goals and received the intervention via the app (an iterated version of the app examined in Mertens et al [46]), (2) a VR condition in which participants set goals and received the intervention via immersive VR, and (3) an active control condition in which participants set goals—as in the other conditions—but received no further intervention.

In all 3 conditions, participants started out with an intake session at the faculty’s research laboratory. During the intake, participants set personal goals and completed a baseline questionnaire. Participants completed online questionnaires during intake (T1 or baseline), at weekly intervals during the intervention (T2 and T3), immediately after the intervention (T4), and 3 and 6 months after the end of the intervention (T5 and T6), or at parallel time points in the control condition. Questionnaires not completed in time (ie, within 4 days for T2 and T3, 8 days for T4, 16 days for T5, and 32 days for T6) were treated as missing data. Data were collected between October 2022 and January 2024.

### Avatar Creation

In both the smartphone and the VR conditions, avatars representing participants’ 10-year older self, that is, the future self, were created with multiple plug-in services and software specifically developed for the research project (all custom software development, design, and illustrations by Orb Amsterdam and Studio Barbaar). The avatar creation process differed slightly between conditions. In the smartphone condition, participants took a “selfie” using the integrated camera of their own smartphone when opening the FutureU app for the first time. This photo was age-progressed by 10 years using a custom-made server and the online service of ChangeMyFace. Subsequently, the aged image was converted into a 3D digital representation via software developed by Avatar SDK, version “Head 2.0.”

In the VR condition, a (full body) avatar was created at the start of the first VR session (T2). This was a 2-step procedure. First, a photo of the participant’s face was made using a webcam that was connected to the computer running the VR simulation. The rest of the procedure for creating the avatar’s head was identical to the smartphone condition. Second, the avatar’s body was created using custom-made software. The software allows for adjusting the proportions of a generic male or female virtual body using a set of sliders to match the participant’s actual body and skin color. The upper-body clothing of the present self-avatar and of the future self-avatar differed in color to emphasize the difference between the 2.

### Participants and Recruitment

Research suggests that the most effective time to intervene is during transformational events and contextual shifts [47]. Such transitions typically involve changes in environment, routines, and social networks. Therefore, we evaluated the effectiveness of the intervention among first-year university students—a group undergoing transition from secondary school to university. This period often involves impactful changes, such as moving away from home, relocating to a new city, and adopting new roles, making it an opportune moment for intervention.

Participants were 321 first-year university students in the Netherlands who were predominantly enrolled in pedagogical sciences (n=187, 58%) and psychology (n=133, 41%). Most participants were female (n=287, 89%) with an average age of 19.59 (SD 2.25) years. Each of the 3 conditions consisted of 107 participants. There were no differences between the conditions regarding sex distribution (*χ*^2^_2_=1.1; *P*=.59; *φ*=.057; female: smartphone: n=97, VR: n=93, control: n=97) and age (*F*_2,318_=0.55; *P*=.58; η^2^_partial_=0.003; smartphone: mean 19.40, SD 2.46; VR: mean 19.70, SD 2.02; control: mean 19.65, SD 2.24).

### Randomization and Allocation Concealment

Participants were randomly assigned to the conditions on a 1:1:1 ratio with blocks of 9. The random sequence was generated with an online tool by the project manager. Participants signed up via the university’s online portal for study participation and were assigned to a condition in the order of enrollment by the project manager in accordance with the generated random sequence. Allocation was known to the researcher at the start of the intake, given that the intake procedure slightly differed between conditions (ie, installing the app and scheduling the VR sessions). Participants were unaware of the different conditions, the intervention’s content, and the study hypotheses.

### Intervention and Comparator

#### Overview

Participants started with setting personal goals; one for the coming year and another one for the coming month. Additionally, they set a goal for the upcoming week as a first step toward reaching their monthly goal. The researcher supported the formulation of these goals and ensured that goals set were specific, measurable, and challenging (though attainable) following the SMART-goal model and Zimmerman’s criteria [48]. In the smartphone and control conditions, participants independently set weekly goals during each week of the intervention period to attain their monthly goal. In the VR condition, participants received additional guidance for setting the weekly goals from the experimenter at the start of each VR session. After setting their goals, participants in the control condition received no further support to achieve their goals. Participants in the intervention conditions received the intervention.

#### Intervention Conditions

After setting their goals, participants received 3 consecutive intervention modules. The first module primarily focused on increasing vividness of the future self, the second module aimed to encourage future-oriented decision-making, and the third module was directed at goal achievement. The theoretical foundation of the 3 modules is presented in Table 1 (see Mertens et al [45] for more information). During development, these modules went through extensive user tests involving both experts and the target population, and a pilot randomized controlled trial [46], to gather qualitative and quantitative feedback for improvements.

#### Smartphone Condition

**Figure 1.**
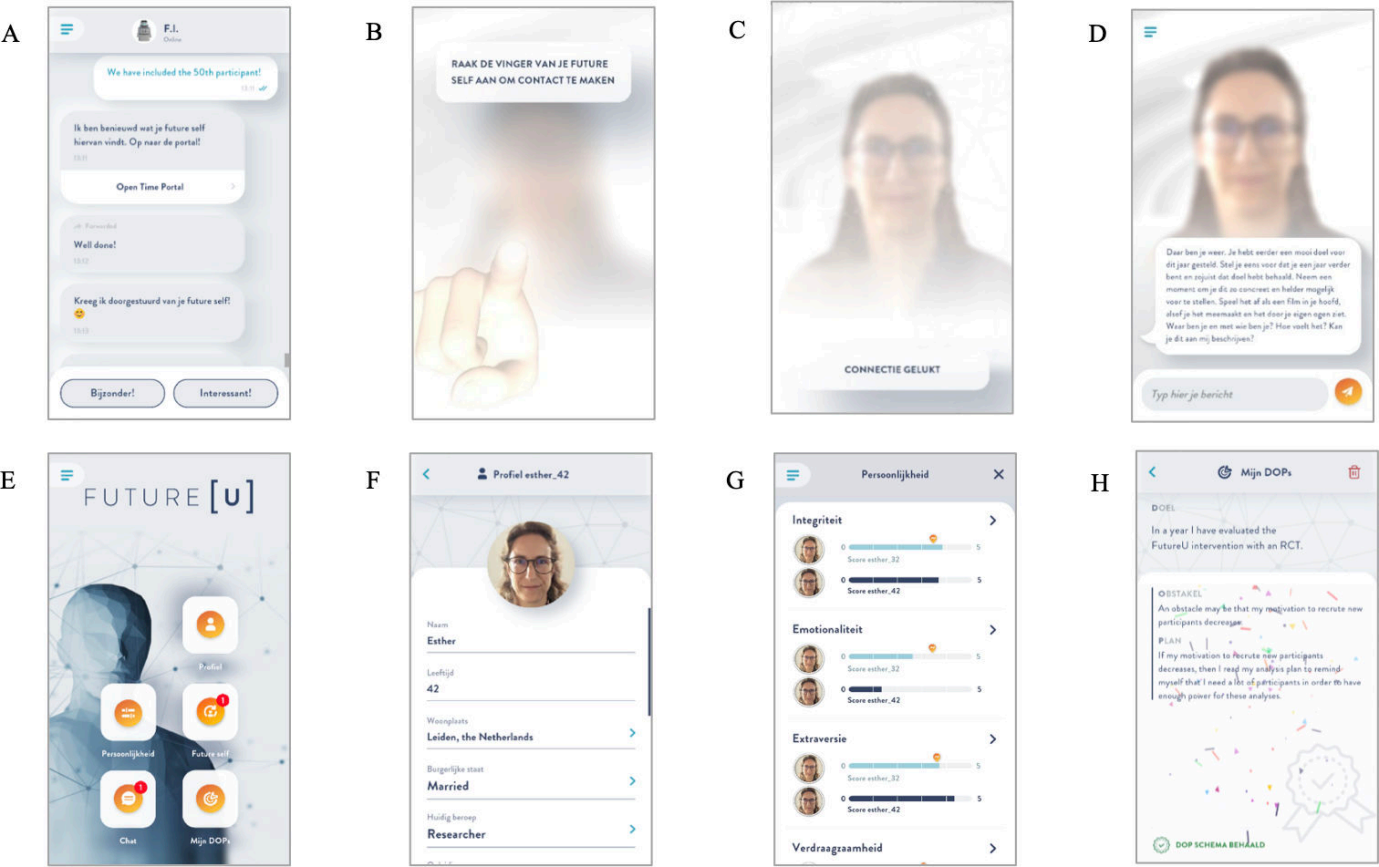
Screenshots of the FutureU smartphone app: (A) chat, (**B**) the connection mechanic, (**C**) future self-interaction, (**D**) home screen, (**E**) personal profile, (**F**) personality menu, and (**G**) goal scheme.

In the app (see Figure 1 for screenshots; reprinted from protocol paper of the current research project [45]), participants were asked to interact with the app on a daily basis for a period of 3 weeks (ie, 21 days) for approximately 5 minutes a day. As a reminder to interact with the app, participants received push notifications. When opening the app, they were directed to the chat feature where they interacted with a chatbot (Figure 1A). This chatbot provided psychoeducation, asked targeted questions, and gave instructions for the interaction or assignment of that day.

Roughly every other day, the app sent an additional push notification from the future self. This notification directed participants to a “future self-interaction” feature. After “connecting” with their future self by touching the virtual finger of their future self (Figure 1B), the screen unblurred, and an interaction with their future self-avatar started (Figure 1C). The consecutive future self-interactions aimed to instill a more vivid image of the future self, encourage participants to think about their future self in their daily life, and stimulate a sense of connection with the future self.

#### VR Condition

**Figure 2.**
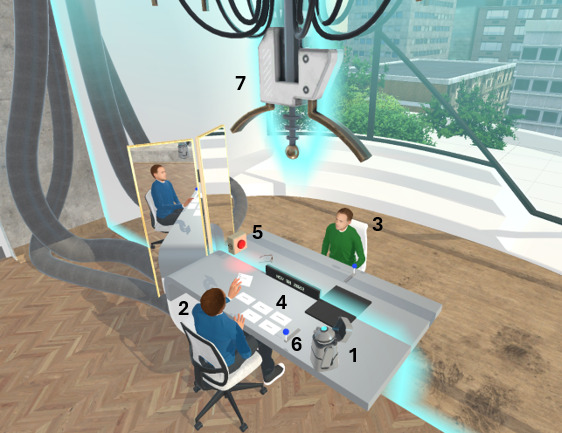
General overview of the FutureU VR environment. 1: robot FI controlled by researcher; 2: present self-avatar; 3: future self-avatar; 4: cards with questions; 5: recording light; 6: handle to travel through time; 7: time machine; VR: virtual reality.

The VR intervention consisted of 3 approximately 30-minute sessions scheduled 1 week apart from each other. The virtual environment consisted of a room with a table and a time machine allowing for “time travel” (Figure 2). Sitting on opposite sides of the table were 2 avatars, one representing the present self, the other representing the future self. The interaction was guided by a researcher-controlled, hovering robot (Figure 2; reprint from protocol paper [45]). This robot provided psychoeducation and explained the controls and interactions to the participants.

During the VR sessions, participants alternated between embodying their present self- and their future self-avatar. While embodying the present self, there were multiple cards with questions lying in front of the participant. They were instructed to read the questions out loud one by one. This process was recorded and, after they finished reading them, they pulled a virtual lever to activate the time machine in order to “time travel” to the future. Following the time travel, they embodied their future self-avatar seated on the other side of the table. The recorded questions were then played back one by one and the participant was invited to respond to them. Again, responses were recorded and, after traveling back to the present, played to the participant who once again was now embodied as their present self. In each session, the first 2 rounds of interaction between the present and future self-avatars were structured using cards. In the third round, participants were free to ask their own questions to their future self. At the end of each session, participants reflected on their answers, which were shown on a floating grid completed by the researcher during the session (The virtual environment was developed with the Unity Pro engine. An HTC Vive head-mounted display with a 110° field-of-view and a resolution of 1080×1200 pixels per eye displayed at 90 Hz was used to deliver the experience. Participants used 2 controllers to move the arms and torso of their avatar.).

### Outcomes

#### Proximal Outcomes

Future self-identification, ie, the extent to which people identify with their future self, was assessed with 3 subscales: vividness, valence, and connectedness. These were assessed at each time point.

Vividness of the future self was assessed with 5 items (eg, “I have a clear image of myself in 10 years from now.”) on a 7-point Likert scale (1=completely disagree to 7=completely agree; T1-T6 α=.91-.93) based on Van Gelder et al [10].

Valence of the future self was measured with a single item: “How do you feel when you think about your future?” [49], answered with the 9-point Self-Assessment Manikin (1=negative to 9=positive).

Connectedness to the future self was measured with the 2-item Future Self-Continuity Measure [49]. These items each consist of a set of 7 increasingly overlapping circles representing the present and the future self. Reliability was adequate at T2 through T6 (α=.70-.79), but somewhat low at T1 (α=.54).

#### Distal Outcomes

##### Primary Outcomes

Future orientation is assessed with the Future Orientation Scale [3] and the Consideration of Future Consequences scale [50]. This scale contains 3 subscales, namely time perspective, anticipation of future consequences, and planning ahead. The scale contains 15 items that each consist of a present-oriented and a future-oriented statement (eg, “Some people spend very little time thinking about how things might be in the future, but other people spend a lot of time thinking about how things might be in the future.”). When choosing the present-oriented statement, participants score a 1=completely true or 2=a little bit true. When choosing the future-oriented statement, participants score a 3=a little bit true or a 4=completely true. This measurement was assessed at all time points (T1-T6: α=.74-.87). At T2 and T3, a selection of 6 items based on factor loadings and face validity were used.

Consideration of future consequences, that is, the degree to which people take immediate versus distant consequences into account in their potential behaviors, was measured by the Consideration of Future Consequences scale by Strathman et al [50]. The scale contained 9 items (eg, “I consider how things might be in the future.”) answered on a 5-point Likert scale (1=completely disagree to 5=completely agree). It was assessed at T1 and T4-T6 (α=.79-.82).

Self-defeating behavior concerns behaviors with immediate gains but potential long-term costs. These were assessed with 15 items (based on Van Gelder et al [10]) assessing self-defeating behaviors (eg, “How often in the last week have you missed classes or work?”) on a 5-point scale (1=never to 5=more than 10 times). Prior research has indicated higher reliability and validity and lower skewness of dichotomized variety scales compared to ordinal frequency scales [51,52]. We therefore dichotomized responses to 0=never and 1=at least once and subsequently summed them to form a scale. Self-defeating behavior was assessed at all time points (T1-T6: α=.60-.68).

Goal commitment to the yearly goal set by the participants was assessed with the Goal Commitment questionnaire [53] consisting of 7 items (eg, “I think this goal is a good goal to shoot for.”) answered on a 7-point Likert scale (1=completely disagree to 7=completely agree). Reliability was good at T4 through T6 (α=.73-.82), though somewhat low at T1 (α=.56).

Weekly and monthly goal achievement measured the degree to which participants reached their weekly and monthly goals. Both were assessed with 3 items on a 5-point Likert scale (1=completely disagree to 5=completely agree) developed for this study: “I have often thought about my goal,” “I have worked hard towards my goal,” and “I have achieved my goal.” Weekly goal achievement was assessed after each intervention week at T2, T3, and T4 (α=.72-.79). Monthly goal achievement was assessed immediately after the intervention at T4 (α=.75).

##### Secondary Outcomes

Self-efficacy assesses the degree to which people feel competent to deal with life’s stressors effectively and was measured with the General Self-Efficacy questionnaire [54] consisting of 10 items (eg, “I can always manage to solve difficult problems if I try hard enough.”) answered on a 4-point Likert scale (1=completely disagree to 4=completely agree). It was assessed at T1 and T4 through T6 (α=.79-.81).

The average academic results of the participants were obtained from the university’s records at the end of the academic year. Impulsiveness was assessed with the Barratt Impulsiveness Scale short form [55]. This questionnaire consists of 10 items (eg, “I do things without thinking.”) answered on a 4-point Likert scale (1=completely disagree to 4*=*completely agree) and was assessed at T1 and T4 through T6 (α=.84-.86).

### Statistical Methods

The data were analyzed with an intention-to-treat approach, in which all participants assigned to the intervention were included in the analyses regardless of whether they received the intervention or not. To include all participants in the analyses, we used full information maximum likelihood procedures with maximum likelihood with robust standard errors estimation. Interim analyses and stopping guidelines were not applied.

The effectiveness of the intervention was examined using a series of latent growth curve (LGC) models in R with the *LAVAAN* package [56]. LGC models estimate an individual growth curve for each participant based on their initial level (ie, intercept) and change over time (ie, slope). These individual growth curves are used as indicators of latent variables that describe the average growth trajectories of the group while allowing for differences in trajectories between participants (when the variance of a slope is fixed to 0, the average growth trajectories of the group are modeled, but not the differences in trajectories between participants) [57]. Intervention effects show when the slope alters in the desired direction compared to the control condition.

In this study, growth rates of slopes were specified to indicate the differences in time intervals between the time points (ie, number of weeks since baseline, including the 3- and 6-month follow-ups), that is, 0, 1, 2, 3, 15, and 27. We modeled 3 different types of LGC models, as not all outcomes were assessed at each time point. For outcomes measured at all time points (ie, T1-T6), we modeled linear piecewise LGC models with a slope for change over time during the intervention (T1-T4) and a slope for change over time during the follow-up period (T4-T6) for each outcome variable separately. For outcomes measured at baseline, immediately after the intervention, and at follow-up (T1, T4-T6), we modeled LGC models, in which T4 was unspecified to allow for nonlinear growth. For weekly goal achievement, measured at T2, T3, and T4, we modeled an LGC model, in which T3 was unspecified. Monthly goal achievement and academic results were assessed at only 1 time point and therefore analyzed with an ANOVA. Sensitivity analyses were conducted to test the robustness of the results (Multimedia Appendix 1). No mediation analyses were conducted, as these were beyond the scope of the paper.

The effects of the intervention were assessed by creating 2 dummy variables (ie, smartphone and VR condition) with the control condition as a reference group and regressing the intercept and the slopes on these 2 dummy variables. In case of intervention effects in the piecewise LGC models, the effect sizes were calculated using the formula: *d*=((*b*_slope 1_*duration_phase 1_)+(*b*_slope 2_*duration_phase 2_))/SD_outcome T1 pooled_. In case of intervention effects in the unconditional LGC models, we used the formula: *d*=(*b*_slope_*duration_Total_)/SD_outcome T1 pooled_ [58]. Additionally, when intervention effects appeared for both intervention conditions, these 2 conditions were subsequently compared by selecting the participants in the intervention conditions and rerunning the model with the smartphone condition as the reference group.

### Ethical Considerations

The study was approved by the independent ethics board of the Institute of Education and Child Studies at Leiden University (ECPW2021-320) and was prospectively registered on ClinicalTrials.gov (NCT05578755) on October 13, 2022, with first participant enrollment on October 19, 2022 (see Mertens et al [45] for the study protocol). No changes to the trial were made after registration. The study is reported following the CONSORT (Consolidated Standards of Reporting Trials) 2025 statement [59] and CONSORT-EHEALTH (Consolidated Standards of Reporting Trials of Electronic and Mobile Health Applications and Online Telehealth) guidelines [60]. All participants provided active informed consent, after reading the information letter and asking any questions they had, at the beginning of the intake before any data were collected. To ensure the privacy of the participants, data were handled according to the study’s Data Management Plan and Data Processing Inventory Analysis, approved by the data manager and privacy officer of Leiden University. Data were anonymized and stored on the secured servers of Leiden University, and access to the data was limited to the research team. The pictures of the participants’ faces were automatically, and permanently, deleted from the secured servers after 24 hours. Participants received either 8 course credits or approximately US $40 for completing the questionnaires of T1 to T4. For completing both follow-up questionnaires (T5 and T6), they received an additional approximately US $23. Figure 1 presents screenshots of the app, showing the future self-rendering of EM, coauthor of this paper. Consent has been granted for the publication of these images.

### Attrition Analyses

In total, 10% of the data were missing. Results of the attrition analyses were based on an α level of .01 to correct for multiple testing. A series of Little’s missing completely at random tests indicated that there was no strong evidence against missing completely at random, though note that one *P* value was .04, which would be considered significant at an α level of .05 (*χ*^2^_2_=0.3‐13.5; *P*=.04-.86).

Attrition analyses showed that participants with missing data at T3 (n=13; multivariate analysis of variance [MANOVA] *F*_9,311_=1.08; *P*=.38; η^2^_partial_=0.030), T4 (n=10; MANOVA *F*_9,311_=1.85; *P*=.06; η^2^_partial_=0.051), T5 (n=67; MANOVA *F*_9,311_=1.78; *P*=.07; η^2^_partial_=0.049), and T6 (MANOVA *F*_9,311_=2.31; *P*=.02; η^2^_partial_=0.063) did not significantly differ at baseline from participants with complete data at the corresponding time point on age and the outcome variables. At T2, there were differences (n=10; MANOVA *F*_9,311_=2.60; *P*=.007; η^2^_partial_=0.070) with participants with missing data scoring lower at baseline on valence (mean 5.70, SD 1.83) than participants with no missing data (mean 6.84, SD 1.21). There were no differences in sex distribution between participants with and without missing data, except at T6. At T6, participants with missing data (n=88) were more often male (*χ*^2^_1_=7.4; *P*=.007; *φ*=−0.152; n=16, 18% vs n=18, 8%).

### Intervention Delivery

In the smartphone condition, treatment adherence was moderate. During the 21-day intervention period, participants were asked to check into the app daily. In total, 12 (11%) participants checked in each day, and 53 (50%) participants checked in on 16 or more days. A total of 17 (16%) participants checked in on 7 or fewer days. Treatment adherence decreased during the intervention. During module 1, a total of 44 (41%) participants checked in daily; during module 2, a total of 30 (28%) participants did so; and during module 3, a total of 18 (17%) participants did so. The number of participants who checked in at least 5 of the 7 days of a module was 91 (85%), 60 (56%), and 52 (49%) respectively. On average, participants used the app for 14 (SD 5.44) days, with 5.56 (SD 1.75) minutes spent on the app on average per day. Furthermore, 13 (4%) participants experienced technical problems with the app, of which 1 (0.3%) was not able to install it. No adverse events or discomfort were reported.

In the VR condition, treatment adherence was adequate; all participants received the 3 VR sessions, except for 3 participants (1 VR session: n=2; 2 VR sessions: n=1). The sessions were intended to be scheduled between 6 and 10 days after intake or the preceding session. In total, 3 (3%) participants did not receive all 3 VR sessions. Of all sessions, 1 (0.3%) was scheduled with a shorter time span between 1 of the 3 sessions, and 43 (14%) were scheduled with a longer time span between 1 of the 3 sessions. The longer time spans ranged from 11 to 14 days between the sessions (n=32, 10%) to 15 to 18 days between the sessions (n=9, 3%). One participant had more than 19 days between sessions 2 and 3. Furthermore, 17 (16%) participants experienced technical problems, such as a distorted voice, during 1 or more sessions. After each VR session, participants were asked by the researcher how they were feeling as well as reported on cybersickness. No adverse events or discomfort were reported.

## Results

### Descriptives and Flowchart

**Figure 3.**
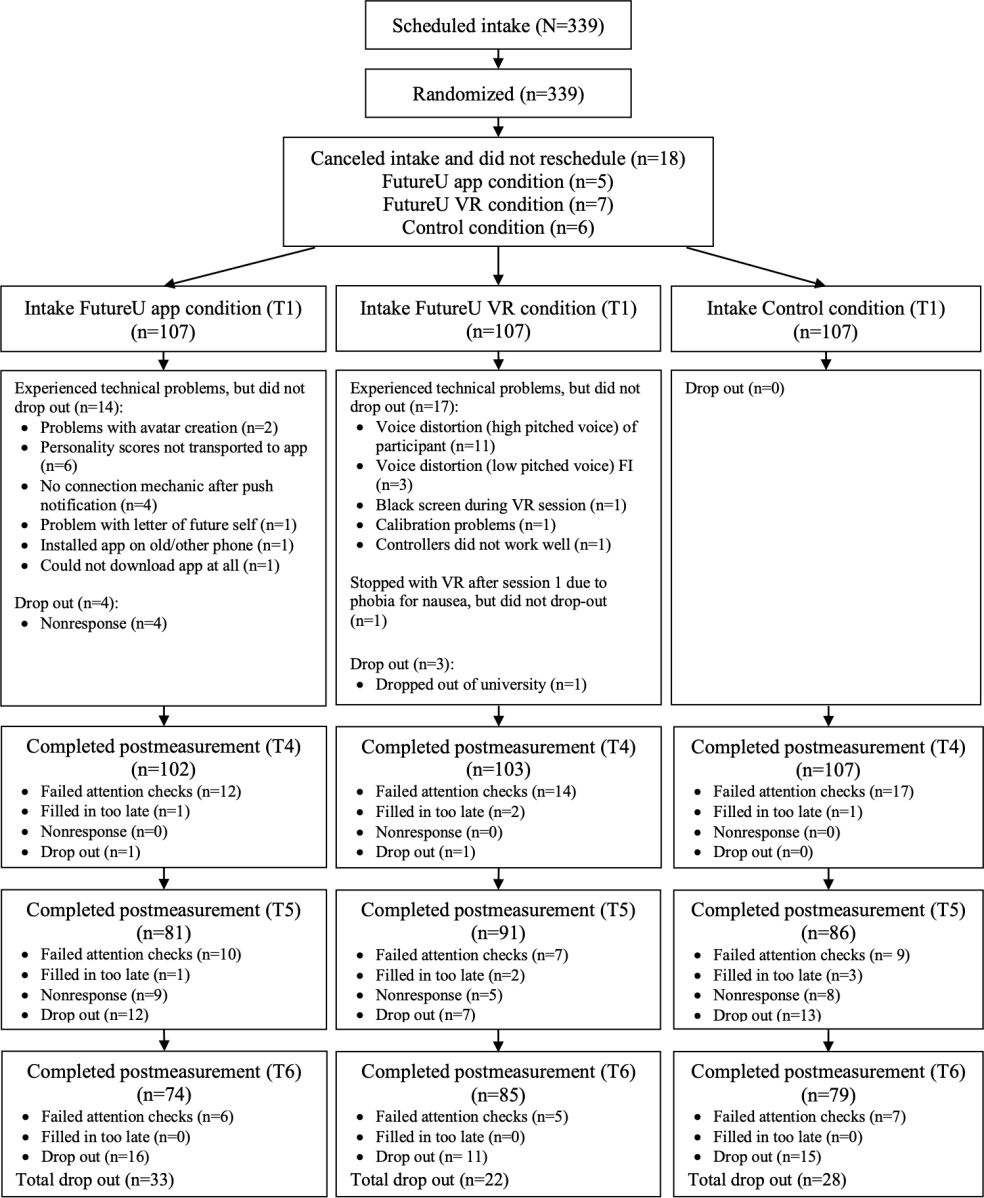
CONSORT flow diagram showing participant recruitment, randomization into the 3 conditions (smartphone, VR, and control), and retention across all 6 time points (from baseline to follow-up). CONSORT: Consolidated Standards of Reporting Trials; VR: virtual reality.

The descriptives of the outcomes per condition at the different time points are presented in Multimedia Appendix 2, and the model fit statistics of the (different types of) LGC models are provided in Table 2. See Figure 3 for flowchart.

**Table 2.** Model fit statistics (CFI^a^, RMSEA^b^, and SRMR^c^) of the latent growth curve models of the outcome variables.

	CFI	RMSEA	SRMR
Proximal outcomes			
Vividness	0.971	0.079	0.036
Valence	0.996	0.022	0.024
Connectedness	0.985	0.055	0.042
Distal outcomes			
Primary outcomes			
Future orientation	0.969	0.095	0.047
Consideration of future consequences	0.996	0.032	0.025
Self-defeating behavior	0.964	0.072	0.038
Goal commitment	0.827	0.148	0.064
Weekly goal achievement^d^	0.915	0.126	0.053
Secondary outcomes			
Self-efficacy^d^	1.000	0.000	0.021
Impulsiveness^e^	1.000	0.010	0.015

a CFI: comparative fit index.

b RMSEA: root mean square error of approximation.

c SRMR: standardized root mean square residual.

d Variance S1 constrained to 0 due to small negative variances.

e Unspecified T4 growth model did not converge, so modeled a linear model with time specified as 0, 3, 15, and 27.

### Intervention Effects on Proximal Outcomes

During the intervention period, the LGC models showed moderate positive intervention effects on all 3 of the proximal outcomes (Table 3). Participants in both the smartphone and the VR conditions increased more on vividness, connectedness, and valence than participants in the control condition. At follow-up, there was a negative effect on vividness and connectedness, in which participants in both intervention conditions decreased more than participants in the control condition. Valence remained stable at follow-up. The effect sizes of the decrease during the 6-month follow-up period were small to moderate and smaller than the increase during the 3-week intervention period. The intervention effects did not differ significantly nor relevantly between the 2 intervention conditions.

**Table 3.** Parameter estimates and effect sizes of the smartphone and virtual reality (VR) conditions compared to the control condition^a^.

	Smartphone condition				VR condition			
	B (SE)	95% CI	*P* value	*d*	B (SE)	95% CI	*P* value	*d*
Proximal outcomes								
Vividness								
I^b^	−0.02 (0.18)	−0.37 to 0.34	.92	—^c^	0.24 (0.17)	−0.10 to 0.57	.17	—
S1^d^	0.23 (0.05)	0.14 to 0.32	<.001	0.49	0.17 (0.05)	0.07 to 0.26	<.001	0.35
S2^e^	−0.02 (0.01)	−0.03 to −0.01	<.001	−0.36	−0.01 (0.01)	−0.02 to −0.00	.01	−0.23
Valence								
I	−0.11 (0.16)	−0.43 to 0.20	.48	—	−0.06 (0.15)	−0.36 to 0.23	.68	—
S1	0.18 (0.05)	0.09 to 0.28	<.001	0.44	0.18 (0.05)	0.09 to 0.28	<.001	0.44
S2	−0.01 (0.01)	−0.02 to 0.00	.17	−0.17	−0.01 (0.01)	−0.02 to 0.01	.27	−0.13
Connectedness								
I	−0.06 (0.12)	−0.30 to 0.18	.62	—	0.14 (0.12)	−0.10 to 0.39	.25	—
S1	0.13 (0.04)	0.06 to 0.21	.001	0.43	0.13 (0.04)	0.05 to 0.21	.001	0.43
S2	−0.01 (0.01)	−0.02 to −0.00	.009	−0.36	−0.01 (0.01)	−0.02 to −0.00	.02	−0.32
Distal outcomes								
Primary outcomes								
Future orientation								
I	0.05 (0.07)	−0.09 to 0.18	.51	—	0.07 (0.07)	−0.06 to 0.21	.29	—
S1	0.03 (0.01)	0.00 to 0.05	.048	0.16	0.03 (0.01)	0.01 to 0.05	.01	0.18
S2	−0.00 (0.00)	−0.01 to −0.00	.01	−0.18	−0.01 (0.00)	−0.01 to −0.00	<.001	−0.25
Consideration of future consequences								
I	0.02 (0.09)	−0.16 to 0.20	.83	—	0.13 (0.08)	−0.02 to 0.28	.09	—
S1	−0.01 (0.00)	−0.01 to 0.00	.29	−0.21	−0.00 (0.00)	−0.01 to 0.01	.55	−0.12
Self-defeating behavior								
I	−0.19 (0.24)	−0.65 to 0.28	.43	—	−0.24 (0.25)	−0.74 to 0.26	.36	—
S1	0.01 (0.08)	−0.15 to 0.17	.93	−0.02	0.04 (0.08)	−0.12 to 0.20	.64	−0.07
S2	0.01 (0.01)	−0.01 to 0.03	.52	−0.08	−0.00 (0.01)	−0.02 to 0.02	.79	0.04
Goal commitment								
I	0.09 (0.07)	−0.05 to 0.23	.20	—	0.01 (0.07)	−0.13 to 0.14	.92	—
S1	−0.00 (0.01)	−0.01 to 0.01	.85	−0.06	−0.00 (0.01)	−0.01 to 0.01	.54	−0.17
Weekly goal achievement								
I	0.10 (0.11)	−0.12 to 0.32	.38	—	0.00 (0.10)	−0.20 to 0.20	.99	—
S1	−0.03 (0.07)	−0.17 to 0.11	.63	−0.08	0.37 (0.08)	0.20 to 0.53	<.001	0.88
Secondary outcomes								
Self-efficacy								
I	0.02 (0.04)	−0.07 to 0.10	.73	—	0.09 (0.05)	−0.01 to 0.18	.08	—
S1	−0.00 (0.00)	−0.01 to 0.00	.43	−0.11	−0.00 (0.00)	−0.01 to 0.00	.19	−0.17
Impulsiveness								
I	−0.01 (0.05)	−0.11 to 0.09	.84	—	−0.06 (0.05)	−0.17 to 0.04	.25	—
S1	0.00 (0.00)	−0.00 to 0.00	.40	−0.07	0.00 (0.00)	−0.00 to 0.00	.27	−0.09

a Positive effect sizes indicate changes in the desired direction.

b I: intercept latent growth curve model.

c Not applicable.

d S1: slope 1 latent growth curve model.

e S2: slope 2 latent growth curve model.

### Intervention Effects on Distal Outcomes: Primary Outcomes

During the intervention period, there was a small positive intervention effect on future orientation. Participants in both intervention conditions remained stable on future orientation, whereas participants in the control condition decreased on this outcome. At follow-up, there was a small negative effect on future orientation for the smartphone and VR conditions compared to the control condition. Participants in the intervention conditions remained stable on future orientation, where participants in the control condition showed a small increase. The effect sizes of the positive effects during the intervention period were almost equal to the effect sizes of the negative effects at follow-up. These effects were similar in both intervention conditions.

There was also a large positive effect on weekly goal achievement in the VR condition compared to the control condition. Participants in the VR condition increased more on weekly goal achievement than participants in the control condition.

No significant nor relevant intervention effects on the other primary outcomes emerged (Table 3). Note, however, that the model fit of the LGC model of goal commitment was moderate (Table 2), which may have reduced sensitivity for significant differences between conditions. Furthermore, there was no intervention effect on monthly goal achievement (ANOVA *F*_1,308_=0.13; *P*=.72; η^2^_partial_=0.00).

### Intervention Effects on Distal Outcomes: Secondary Outcomes

There were no significant or relevant intervention effects on the secondary outcomes (Table 3). In addition, there was no effect on academic results (ANOVA *F*_1,316_=1.02; *P*=.31; η^2^_partial_=0.00).

## Discussion

### Summary of Main Findings

This study provides evidence that future-oriented thinking and goal achievement can be strengthened through a digital intervention focused on fostering identification with the future self. Consistent with expectations, the intervention showed robust effects on proximal outcomes, with both delivery modes significantly increasing vividness of, connectedness with, and valence toward the future self during the intervention period. At follow-up, the intervention effect on valence remained stable, whereas a partial reversal of the effects on vividness and connectedness was observed. The finding that intervention effects slightly decreased over time, albeit less than the initial increase, indicates that the gains in future self-identification may attenuate over time without continued reinforcement. This suggests that booster sessions or continued exposure to the future self could be necessary to maintain the effects. An intervention app may be best suited for this, as an app can remain installed on participants’ smartphones after the intervention has concluded to deliver intervention content, eg, push notifications and/or future self-avatar messages, or allow participants to use the app on their own initiative [32,33].

The intervention had more limited effects on the distal outcomes, although there was a small positive effect on future orientation in both intervention conditions and a strong positive effect on weekly goal achievement when the intervention was delivered via VR. There was no effect on monthly goal achievement, indicating that short-term goal engagement may have been boosted but not more durable goal progress. No effects were found for the other primary outcomes (ie, consideration of future consequences, self-defeating behavior, goal commitment, and monthly goal achievement) nor for the secondary outcomes (ie, self-efficacy, impulsiveness, and academic performance).

The positive effect on future orientation may indicate that enhanced future self-identification indeed cultivates a future-oriented mindset, as theorized [10,12]. However, more distal and often more deeply embedded behavioral patterns related to such a mindset, such as self-defeating behavior, may need a stronger, more focused, and/or more sustained intervention. Importantly, these null findings were consistent across measures and time points, suggesting that while the intervention momentarily influenced self-perception and weekly goal engagement within the intervention context, such changes did not translate into sustained or generalized behavioral change over the longer term, signaling room for further optimization.

Regarding weekly goal achievement, the intervention had no effect when delivered via the app, but there was a strong effect when delivered in VR. The VR intervention created an immersive experience, in which people talked to their future self, embodied this self, and took its perspective. As such, the VR condition created a first-person experience of how it could feel to have achieved personal goals. Imagination of outcomes has been shown to be an important step toward goal achievement [61]. Nonetheless, the difference between delivery modes cannot be attributed unambiguously to immersive VR technology per se. The VR condition differed from the smartphone condition on several dimensions beyond immersion that may have also affected goal achievement. Specifically, because FutureU VR was provided in person, people in the VR condition had direct interaction with a researcher and received explicit checks of weekly goals against SMART criteria. In contrast, participants using the app engaged independently with brief daily app interactions, without personal contact, external accountability, or synchronous feedback on SMART criteria. The possibility that these differences had effects over and above the immersion in VR can therefore not be excluded. As such, the observed advantage of the VR condition should be interpreted as hypothesis-generating rather than as evidence that immersive VR delivery is inherently more effective than app-based delivery for goal achievement.

Taken together, these results underscore a key design challenge for future self-interventions: brief, standalone VR experiences and app interactions can be effective for eliciting short-term psychological shifts, but driving enduring behavioral or academic change can be more challenging. Rather than a limitation unique to this study, the absence of distal effects aligns with broader evidence that translating changes in future-oriented thinking into lasting real-world behavior likely requires repeated exposure, scaffolding, and integration with ongoing self-regulatory support [33]. As such, the present findings contribute to a more nuanced understanding of what VR- and app-based future self-interventions can—and cannot—be expected to achieve in the short term.

### Strengths and Limitations

A key strength of this study is its rigorous randomized controlled design and relatively large sample size. Prior work on future self-identification has often relied on cross-sectional data and/or smaller samples (for exceptions, see Hershfield et al [14] and Ganschow et al [62]). Additionally, the inclusion of both a smartphone and a VR modality enabled direct comparison of these delivery formats. Furthermore, research on future self-identification has generally targeted only one of the aspects of its tripartite structure or used index measures that do not allow for disentangling individual effects [33], whereas our study included all three simultaneously and examined them separately.

Several limitations of this study must also be acknowledged. First, and foremost, delivery modality was confounded with other design features. The VR condition involved in-person researcher involvement and structured goal review, whereas the smartphone condition relied on brief, self-guided engagement. While more extensive personal contact can be seen as a secondary characteristic of this type of technology, our design makes it difficult to isolate the specific contribution of immersive VR technology from the effects of human support. To disentangle these factors, future research could, for example, provide equivalent in-person guidance across smartphone and VR conditions or test fully automated VR interventions without researcher involvement. Such designs would allow clearer conclusions about whether embodiment and immersion independently contribute to goal achievement.

Second, although we deliberately opted for first-year university students as this sample allowed us to capitalize on a major life transition phase, which may increase receptivity to interventions, it also limits the generalizability of our findings. The sample was predominantly female and drawn from a relatively narrow university context, a population that may already be relatively future-focused and hence have limited room for growth. Additionally, participants received compensation for completing the questionnaires, which could have affected the results and limited generalization to uncompensated populations. Furthermore, self-reported measures, especially for constructs like self-defeating behavior and impulsivity, may be susceptible to bias or lack of sensitivity to subtle behavioral changes, which may therefore not have been picked up. Finally, some effects may have been masked by measurement timing; for instance, positive gains in future orientation during the intervention period may have waned by follow-up, while longer-term benefits (eg, academic performance) may not yet have materialized.

### Conclusions

Given the transdiagnostic potential of the future self—promoting positive behavior [9,13], reducing negative behavior [15,16,19], and improving mental health [20]—it is increasingly leveraged as an intervention mechanism. Building on previous research, this study evaluated a future self-intervention and not only examined all three aspects of future self-identification but also a novel implementation method with new technologies. Where more traditional future self-interventions rely on imagination [30,31], visual and immersive technologies, such as smartphone apps versus VR, can relieve this cognitive burden by presenting graphic renderings of the future self and enabling a sense of interaction with it. The results reported in this study suggest that visual and interactive technologies may serve as promising tools for strengthening future self-identification, cultivating a future-oriented mindset, and contributing to goal achievement. To enhance and sustain effects, future work could explore the integration of booster sessions, more personalized content delivery (eg, through the integration of artificial intelligence–powered conversational agents), application in more diverse and at-risk populations, and more targeted interventions. Indeed, recent work has already started to leverage novel possibilities offered by generative artificial intelligence, including voice cloning and personalized biographical narratives [63-65], to augment future-oriented thinking. All in all, the results of these studies and our own underscore the potential of future self-based interventions using novel technologies as scalable strategies to support long-term goal pursuit and positive psychosocial development.

## Supplementary material

10.2196/84420 Multimedia Appendix 1 Sensitivity analyses.

10.2196/84420 Multimedia Appendix 2 Descriptives of the outcomes per condition per time point.

10.2196/84420 Checklist 1 CONSORT checklist.

10.2196/84420 Checklist 2 CONSORT-eHEALTH checklist (V 1.6.1).

## Abbreviations

CONSORT: Consolidated Standards of Reporting Trials
CONSORT-EHEALTH: Consolidated Standards of Reporting Trials of Electronic and Mobile Health Applications and Online Telehealth
LGC: latent growth curve
MANOVA: multivariate analysis of variance
VR: virtual reality
