# Supplementary material for: Stimulating Future-Oriented Thinking and Goal-Achievement Through the Future Self Using Virtual Reality and a Smartphone App: Randomized Controlled Trial
Source: J Med Internet Res. 2026 May 13;28:e84420. doi: 10.2196/84420 (PMC13170087; doi:10.2196/84420)
Supplement: Multimedia Appendix 1 [file jmir-v28-e84420-s001.docx]

**Sensitivity analyses**

**Analysis Plan**

The robustness of the results of the analyses examining differences in change over time between conditions were examined with sensitivity analyses. In these analyses participants were excluded if 1) they experienced any technological problems with the app or during a VR session, and/or 2) failed one or both attention checks embedded in the post and follow-up measurements (T4, T5, T6). Each of these questionnaires contained two items instructing participants to select a specific response, serving as an indicator of their (in)attentiveness.

**Results**

In total, 14 different participants experienced problems with the app, 15 during at least one VR sessions, 43 failed at least one attention check at T4, 26 at T5, and 18 at T6. In total, 97 participants were excluded for the sensitivity analyses resulting in a total sample of 224 participants. The model fit statistics are reported in Table S1 and the parameter estimates in Table S2.

***Intervention Effects on Proximal Outcomes***

The sensitivity analyses showed roughly the same results as the analyses on the full sample. In both intervention conditions, students increased during the intervention more on vividness, valence, and relatedness than students in the control condition. At follow-up, students in the intervention conditions slightly decreased on vividness and relatedness compared to the control condition. Additionally, students in the smartphone condition slightly decreased on valence at follow-up compared to the control condition, while the results regarding the full sample indicated no differences between students in the smartphone and control conditions.

***Intervention Effects on Primary Outcomes***

The results were generally the same as for the full sample. Students in both intervention conditions increased on future orientation while students in the control condition remained stable. At follow-up, students in the intervention conditions remained stable, whereas students in the control condition slightly increased. Furthermore, students in the VR condition increased more on weekly goal achievement compared to students in the control condition.

Result regarding considerations of future consequences differed from those of the full sample. For this outcome, the unspecified growth model did not converge. Therefore, a linear growth model was specified. This model indicated a small decrease on consideration of future consequences in both intervention conditions compared to the control condition. However, as this model differs from the model used to analyze the full sample, it is not possible to directly compare these results.

***Intervention Effects on Secondary Outcomes***

The results of the sensitivity analyses were the same as those of the full sample: There were no differences in change over time on any of the outcomes between the conditions.

**Table S1.** Model Fit Statistics of the LGC Models of the Outcome Variables of the Sensitivity Sample

|  | | **CFI** | **RMSEA** | **SRMR** |
| --- | --- | --- | --- | --- |
| **Proximal outcomes** | |  |  |  |
|  | Vividness | .971 | .079 | .039 |
|  | Valence | .997 | .019 | .030 |
|  | Relatedness | .986 | .053 | .041 |
| **Distal outcomes** | |  |  |  |
| ***Primary outcomes*** | |  |  |  |
|  | Future Orientation | .969 | .100 | .050 |
|  | Consideration of future consequences^2^ | .990 | .045 | .032 |
|  | Self-defeating behavior | .980 | .055 | .059 |
|  | Goal commitment^1^ | .780 | .158 | .075 |
|  | Weekly goal achievement^1^ | .944 | .105 | .049 |
|  | Monthly goal achievement ^Anova^ |  |  |  |
| ***Secondary outcomes*** | |  |  |  |
|  | Self-efficacy^1^ | 1.000 | .000 | .025 |
|  | Academic results ^Anova^ |  |  |  |
|  | Impulsiveness^2^ | .998 | .027 | .018 |

*Note*. ^1^Variance S1 constrained to 0 due to small negative variances; ^2^Unspecified T4 growth model did not converge, so modeled a linear model with time specified as 0, 3, 15, 27; CFI = Comparative Fit Index; RMSEA = Root Mean Square Error of Approximation; SRMR = Standardized Root Mean Squared Residual

**Table S2.** Parameter Estimates of the Smartphone and VR conditions Compared to the Control Condition

|  | |  |  | **Smartphone condition** | | | | **VR condition** | | | |
| --- | --- | --- | --- | --- | --- | --- | --- | --- | --- | --- | --- |
|  | |  |  | B | SE | p |  | B | SE | p |  |
| **Proximal outcomes** | | |  |  |  |  |  |  |  |  |  |
|  | Vividness | | I | 0.23 | .21 | .280 |  | 0.25 | .20 | .220 |  |
|  |  | | S1 | **0.22** | **.06** | **< .001** |  | **0.23** | **.06** | **< .001** |  |
|  |  | | S2 | **-0.02** | **.01** | **.008** |  | **-0.01** | **.01** | **.032** |  |
|  | Valence | | I | -0.13 | .20 | .522 |  | -0.13 | .18 | .488 |  |
|  |  | | S1 | **0.23** | **.06** | **< .001** |  | **0.22** | **.06** | **< .001** |  |
|  |  | | S2 | **-0.01** | **.01** | **.046** |  | -0.01 | .01 | .467 |  |
|  | Relatedness | | I | 0.02 | .15 | .879 |  | 0.18 | .15 | .221 |  |
|  |  | | S1 | **0.16** | **.05** | **.001** |  | **0.17** | **.05** | **.001** |  |
|  |  | | S2 | **-0.02** | **.01** | **.007** |  | **-0.02** | **.01** | **.007** |  |
| **Distal outcomes** | | |  |  |  |  |  |  |  |  |  |
| ***Primary outcomes*** | | |  |  |  |  |  |  |  |  |  |
|  | Future Orientation | | I | 0.05 | .08 | .569 |  | 0.08 | .08 | .320 |  |
|  |  | | S1 | **0.03** | **.02** | **.032** |  | **0.03** | **.01** | **.022** |  |
|  |  | | S2 | **-0.00** | **.00** | **.025** |  | **-0.01** | **.00** | **.006** |  |
|  | Consideration of fut. Con. | | I | 0.10 | .09 | .285 |  | **0.20** | **.08** | **.016** |  |
|  |  | | S1 | **-0.01** | **.00** | **.016** |  | **-0.01** | **.00** | **.012** |  |
|  | Self-defeating behavior | | I | -0.07 | .27 | .802 |  | -0.11 | .30 | .718 |  |
|  |  | | S1 | -0.02 | .09 | .851 |  | 0.02 | .09 | .865 |  |
|  |  | | S2 | -0.01 | .01 | .623 |  | -0.01 | .01 | .529 |  |
|  | Goal commitment | | I | 0.06 | .08 | .453 |  | -0.04 | .08 | .643 |  |
|  |  | | S1 | 0.00 | .01 | .757 |  | -0.00 | .01 | .734 |  |
|  | Weekly goal achievement | | I | 0.03 | .13 | .796 |  | -0.02 | .12 | .896 |  |
|  |  | | S1 | 0.03 | .08 | .723 |  | **0.36** | **.09** | **< .001** |  |
| ***Secondary outcomes*** | | |  |  |  |  |  |  |  |  |  |
|  | Self-efficacy | | I | -0.04 | .05 | .425 |  | 0.05 | .06 | .347 |  |
|  |  | | S1 | -0.00 | .00 | .983 |  | -0.00 | .00 | .823 |  |
|  | Impulsiveness | | I | -0.03 | .07 | .666 |  | -0.09 | .07 | .171 |  |
|  |  | | S1 | 0.00 | .00 | .347 |  | 0.00 | .00 | .571 |  |

*Note*. I = Intercept LGC model; S1 = Slope 1 LGC model; S2 = Slope 2 LGC model; Significant findings (*p* < .05) are emphasized in bold.
