# Supplementary material for: Stimulating Future-Oriented Thinking and Goal-Achievement Through the Future Self Using Virtual Reality and a Smartphone App: Randomized Controlled Trial
Source: J Med Internet Res. 2026 May 13;28:e84420. doi: 10.2196/84420 (PMC13170087; doi:10.2196/84420)
Supplement: Multimedia Appendix 2 [file jmir-v28-e84420-s002.docx]

**Table S1. Descriptives of the outcomes per condition per time point.**

|  | |  | Smartphone condition | | | | | |  | VR condition | | | | | |  | Control condition | | | | | |
| --- | --- | --- | --- | --- | --- | --- | --- | --- | --- | --- | --- | --- | --- | --- | --- | --- | --- | --- | --- | --- | --- | --- |
|  | |  |  | | | | | |  |  | | | | | |  |  | | | | | |
|  | |  | T1 | T2 | T3 | T4 | T5 | T6 |  | T1 | T2 | T3 | T4 | T5 | T6 |  | T1 | T2 | T3 | T4 | T5 | T6 |
|  | |  | M (SD) | M (SD) | M (SD) | M (SD) | M (SD) | M (SD) |  | M (SD) | M (SD) | M (SD) | M (SD) | M (SD) | M (SD) |  | M (SD) | M (SD) | M (SD) | M (SD) | M (SD) | M (SD) |
| **Proximal outcomes** | | |  |  |  |  |  |  |  |  |  |  |  |  |  |  |  |  |  |  |  |  |
|  | Vividness | | 3.30 (1.45) | 3.99 (1.28) | 4.28 (1.22) | 4.34 (1.27) | 4.09 (1.26) | 3.99 (1.34) |  | 3.67 (1.33) | 4.10 (1.04) | 4.37 (1.15) | 4.46 (1.08) | 4.33 (1.29) | 4.24 (1.30) |  | 3.41 (1.43) | 3.73 (1.32) | 3.75 (1.26) | 3.74 (1.36) | 3.68 (1.32) | 3.94 (1.42) |
|  | Valence | | 6.64 (1.41) | 6.91 (1.14) | 6.88 (1.21) | 6.99 (1.20) | 6.77 (1.26) | 6.84 (1.17) |  | 6.85 (1.20) | 6.80 (1.05) | 6.99 (0.99) | 7.06 (1.07) | 6.92 (1.23) | 6.80 (1.32) |  | 6.91 (1.12) | 6.72 (1.11) | 6.55 (1.16) | 6.59 (1.08) | 6.51 (1.23) | 6.51 (1.18) |
|  | Connectedness | | 3.79 (0.96) | 3.97 (1.00) | 4.21 (1.03) | 4.43 (1.07) | 4.29 (0.90) | 4.33 (1.02 |  | 3.95 (0.95) | 4.19 (0.99) | 4.35 (1.10) | 4.59 (1.08) | 4.57 (1.09) | 4.40 (1.18) |  | 3.80 (0.89) | 3.93 (0.92) | 3.94 (0.98) | 4.05 (0.98) | 4.18 (1.01) | 4.24 (1.02) |
| **Distal outcomes** | | |  |  |  |  |  |  |  |  |  |  |  |  |  |  |  |  |  |  |  |  |
| **Primary outcomes** | | |  |  |  |  |  |  |  |  |  |  |  |  |  |  |  |  |  |  |  |  |
|  | Future Orientation | | 3.07 (0.46) | 3.05 (0.52) | 3.05 (0.49) | 3.06 (0.43) | 3.07 (0.42) | 3.08 (0.41) |  | 3.09 (0.50) | 3.08 (0.54) | 3.12 (0.54) | 3.08 (0.50) | 3.12 (0.46) | 3.09 (0.48) |  | 3.02 (0.49) | 3.00 (0.59) | 2.99 (0.57) | 2.92 (0.49) | 2.99 (0.48) | 3.08 (0.39) |
|  | Consideration of fut. consequences | | 3.50 (0.58) | - | - | 3.56 (0.54) | 3.51 (0.56) | 3.47 (0.50) |  | 3.71 (0.64) | - | - | 3.60 (0.49) | 3.65 (0.49) | 3.64 (0.52) |  | 3.49 (0.55) | - | - | 3.45 (0.55) | 3.57 (0.53) | 3.63 (0.56) |
|  | Self-defeating beh. | | 5.10 (1.71) | 4.46 (1.91) | 4.15 (1.95) | 4.16 (2.02) | 4.77 (2.15) | 4.74 (1.77) |  | 4.97 (1.95) | 4.52 (1.95) | 4.38 (2.08) | 3.96 (1.94) | 4.64 (1.74) | 4.42 (2.02) |  | 5.31 (1.98) | 4.69 (1.87) | 4.14 (1.89) | 4.41 (2.11) | 4.91 (1.01) | 4.69 (1.91) |
|  | Goal commitment | | 6.06 (0.53) | - | - | 5.84 (0.68) | 5.56 (0.80) | 5.46 (1.00) |  | 5.99 (0.49) | - | - | 5.69 (0.70) | 5.38 (1.01) | 5.24 (0.99) |  | 5.99 (0.52) | - | - | 5.71 (0.79) | 5.31 (1.05) | 5.44 (0.99) |
|  | Weekly goal ach. | | - | 3.58 (0.84) | 3.45 (1.05) | 4.06 (1.36) | - | - |  | - | 3.38 (0.80) | 3.40 (0.81) | 4.76 (1.37) | - | - |  | - | 3.49 (0.86) | 3.32 (0.97) | 3.98 (1.19) | - | - |
|  | Monthly goal ach. | | - | - | - | 3.54 (0.90) | - | - |  | - | - | - | 3.51 (0.72) | - | - |  | - | - | - | 3.47 (0.80) | - | - |
| **Secondary outcomes** | | |  |  |  |  |  |  |  |  |  |  |  |  |  |  |  |  |  |  |  |  |
|  | Self-efficacy | | 2.84 (0.33) | - | - | 2.91 (0.33) | 2.93 (0.35) | 2.96 (0.37) |  | 2.91 (0.38) | - | - | 2.96 (0.36) | 2.98 (0.37) | 2.97 (0.34) |  | 2.84 (0.35) | - | - | 2.88 (0.32) | 2.93 (0.31) | 2.94 (0.33) |
|  | Academic results | | - | - | - | - | - | 7.13 (0.59) |  | - | - | - | - | - | 7.13 (0.58) |  | - | - | - | - | - | 7.21 (0.59) |
|  | Impulsiveness | | 2.22 (0.38) | - | - | 2.17 (0.38) | 2.13 (0.39) | 2.18 (0.39) |  | 2.16 (0.40) | - | - | 2.12 (0.37) | 2.13 (0.38) | 2.14 (0.41) |  | 2.22 (0.42) | - | - | 2.20 (0.42) | 2.16 (0.40) | 2.18 (0.40) |

*Note.* Consideration of fut. Consequences = Consideration of future consequences; Self-defeating beh. = Self-defeating behavior; Weekly goal ach. = Weekly goal achievement; Monthly goal ach. = Monthly goal achievement
