# Supplementary material for: Stimulating Future-Oriented Thinking and Goal-Achievement Through the Future Self Using Virtual Reality and a Smartphone App: Randomized Controlled Trial
Source: J Med Internet Res. 2026 May 13;28:e84420. doi: 10.2196/84420 (PMC13170087; doi:10.2196/84420)
Supplement: Checklist 1 [file jmir-v28-e84420-s003.docx]

|  | Section/topic | No | CONSORT 2025 checklist item description | Reported on page no. |
| --- | --- | --- | --- | --- |
|  | **Title and abstract** | | |  |
|  | Title and structured abstract | 1a | Identification as a randomised trial | p1 “A Randomized Controlled Trial” |
|  |  | 1b | Structured summary of the trial design, methods, results, and conclusions | p2 “Background: Research suggests (…) goal pursuit” |
|  | **Open science** | | |  |
|  | Trial registration | 2 | Name of trial registry, identifying number (with URL) and date of registration | p3 “The trial is concluded (…) on 19 October 2022”  p21, “was prospectively registered (…) 19 October 2022” |
|  | Protocol and statistical analysis plan | 3 | Where the trial protocol and statistical analysis plan can be accessed | p3 “the study protocol is published (see [44])”  p21 “(see [44] for the study protocol)” |
|  | Data sharing | 4 | Where and how the individual de-identified participant data (including data dictionary), statistical code and any other materials can be accessed | p31 “Data that support (…) VR-based intervention” |
|  | Funding and conflicts of interest | 5a | Sources of funding and other support (eg, supply of drugs), and role of funders in the design, conduct, analysis and reporting of the trial | p31 “This study was financially (…) writing of the manuscript” |
|  |  | 5b | Financial and other conflicts of interest of the manuscript authors | p31 “The authors declare (…) interests” |
|  | **Introduction** | | |  |
|  | Background and rationale | 6 | Scientific background and rationale | p4-9 “Considering the future (…) were formulated” |
|  | Objectives | 7 | Specific objectives related to benefits and harms | p9 “The goal of this study (…) research program” |
|  | **Methods** | | |  |
|  | Patient and public involvement | 8 | Details of patient or public involvement in the design, conduct and reporting of the trial | p13 “these modules went through extensive user-tests involving both experts and the target population, and a pilot RCT (see [41]), to gather qualitative and quantitative feedback for improvements”  Protocol paper |
|  | Trial design | 9 | Description of trial design including type of trial (eg, parallel group, crossover), allocation ratio, and framework (eg, superiority, equivalence, non-inferiority, exploratory) | p10 “parallel Randomized Controlled Trial” p12 “Participants were randomly (…) and study hypotheses” |
|  | Changes to trial protocol | 10 | Important changes to the trial after it commenced including any outcomes or analyses that were not prespecified, with reason | p21 “No changes to the trial were made after registration” |
|  | Trial setting | 11 | Settings (eg, community, hospital) and locations (eg, countries, sites) where the trial was conducted | p10 “the faculty’s research lab”  p11 “in the Netherlands” |
|  | Eligibility criteria | 12a | Eligibility criteria for participants | p11 “first-year university students in the Netherlands”  Protocol paper |
|  |  | 12b | **If applicable**, eligibility criteria for sites and for individuals delivering the interventions (eg, surgeons, physiotherapists) | N/A |
|  | Intervention and comparator | 13 | Intervention and comparator with sufficient details to allow replication. If relevant, where additional materials describing the intervention and comparator (eg, intervention manual) can be accessed | p12-16 “Participants started with (…) during the session” |
|  | Outcomes | 14 | Prespecified primary and secondary outcomes, including the specific measurement variable (eg, systolic blood pressure), analysis metric (eg, change from baseline, final value, time to event), method of aggregation (eg, median, proportion), and time point for each outcome | p16-19 “Outcomes. proximal outcomes. (…) and T4 through T6 (α = .84-.86)” |
|  | Harms | 15 | How harms were defined and assessed (eg, systematically, non-systematically) | p22 “No adverse events or discomfort were reported”  p23 “After each VR session, (…) were reported” |
|  | Sample size | 16a | How sample size was determined, including all assumptions supporting the sample size calculation | Protocol paper |
|  |  | 16b | Explanation of any interim analyses and stopping guidelines | p19 “Interim analyses and stopping guidelines were not applied” |
|  | Randomisation: |  |  |  |
|  | Sequence generation | 17a | Who generated the random allocation sequence and the method used | p12 “The random sequence was generated with an online tool by the project manager” |
|  |  | 17b | Type of randomisation and details of any restriction (eg, stratification, blocking and block size) | p12 “on a 1:1:1 ration with blocks of 9” |
|  |  |  |  | **Reported on page no.** |
|  | Allocation concealment mechanism | 18 | Mechanism used to implement the random allocation sequence (eg, central computer/telephone; sequentially numbered, opaque, sealed containers), describing any steps to conceal the sequence until interventions were assigned | p12 “Participants signed up (…) and study hypotheses”  Protocol paper |
|  | Implementation | 19 | Whether the personnel who enrolled and those who assigned participants to the interventions had access to the random allocation sequence | p12 “were assigned to (…) scheduling the VR sessions)” |
|  | Blinding | 20a | Who was blinded after assignment to interventions (eg, participants, care providers, outcome assessors, data analysts) | p12 “Allocation was known (…) and study hypotheses”  Protocol paper |
|  |  | 20b | **If blinded**, how blinding was achieved and description of the similarity of interventions | N/A |
|  | Statistical methods | 21a | Statistical methods used to compare groups for primary and secondary outcomes, including harms | p19-21 “The data were analyzed (…) as reference group” |
|  |  | 21b | Definition of who is included in each analysis (eg, all randomised participants), and in which group | p19 “The data were (…) Errors (MLR) errors”  Fig 1 |
|  |  | 21c | How missing data were handled in the analysis | p19 “we used Full Information Maximum Likelihood (…) (MLR) estimation” |
|  |  | 21d | Methods for any additional analyses (eg, subgroup and sensitivity analyses), distinguishing prespecified from post hoc | p20 “Sensitivity analyses (…) Supplementary Materials)” |
|  | **Results** | | |  |
|  | Participant flow, including flow diagram | 22a | For each group, the numbers of participants who were randomly assigned, received intended intervention, and were analysed for the primary outcome | Fig 1 |
|  |  | 22b | For each group, losses and exclusions after randomisation, together with reasons | Fig 1 |
|  | Recruitment | 23a | Dates defining the periods of recruitment and follow-up for outcomes of benefits and harms | p10 “Participants completed (…) January 2024” |
|  |  | 23b | **If relevant**, why the trial ended or was stopped | N/A |
|  | Intervention and comparator delivery | 24a | Intervention and comparator as they were actually administered (eg, where appropriate, who delivered the intervention/comparator, how participants adhered, whether they were delivered as intended (fidelity)) | p22-23 “In the smartphone condition (…) during one or more sessions” |
|  |  | 24b | Concomitant care received during the trial for each group | p12 “After setting their goals, (…) received the intervention” |
|  | Baseline data | 25 | A table showing baseline demographic and clinical characteristics for each group | p12 |
|  | Numbers analysed,  outcomes and estimation | 26 | For each primary and secondary outcome, by group:  ● the number of participants included in the analysis  ● the number of participants with available data at the outcome time point  ● result for each group, and the estimated effect size and its precision (such as 95% confidence interval)  ● for binary outcomes, presentation of both absolute and relative effect size | p24 “Intervention effects on proximal outcomes (…) η^2^_partial_ = 0.00)”  Table 4  Fig 1 |
|  | Harms | 27 | All harms or unintended events in each group | p22 “No adverse events or discomfort were reported”  p23 “No adverse events or discomfort were reported” |
|  | Ancillary analyses | 28 | Any other analyses performed, including subgroup and sensitivity analyses, distinguishing pre-specified from post hoc | p20 “Sensitivity analyses (…) Supplementary Materials)”  p23 “The sensitivity analyses showed (…) Supplementary Materials” |
|  | **Discussion** | | |  |
|  | Interpretation | 29 | Interpretation consistent with results, balancing benefits and harms, and considering other relevant evidence | p26-28 “The present study (…) in the short term” |
|  | Limitations | 30 | Trial limitations, addressing sources of potential bias, imprecision, generalisability, and, if relevant, multiplicity of analyses | p28-29 “A key strength (…) not yet have materialized” |

Citation: Hopewell S, Chan AW, Collins GS, Hróbjartsson A, Moher D, Schulz KF, et al. CONSORT 2025 Statement: updated guideline for reporting randomised trials. BMJ. 2025; 388:e081123. <https://dx.doi.org/10.1136/bmj-2024-081123>
© 2025 Hopewell et al. This is an Open Access article distributed under the terms of the Creative Commons Attribution License (<https://creativecommons.org/licenses/by/4.0/>), which permits unrestricted use, distribution, and reproduction in any medium, provided the original work is properly cited.

*We strongly recommend reading this statement in conjunction with the CONSORT 2025 Explanation and Elaboration and/or the CONSORT 2025 Expanded Checklist for important clarifications on all the items. We also recommend reading relevant CONSORT extensions. See [www.consort-spirit.org](http://www.consort-spirit.org).
